# Positive Deviance for Dual-Method Promotion among Women in Uganda: A Qualitative Study

**DOI:** 10.3390/ijerph17145009

**Published:** 2020-07-12

**Authors:** Hodaka Kosugi, Akira Shibanuma, Junko Kiriya, Ken Ing Cherng Ong, Stephen Mucunguzi, Conrad Muzoora, Masamine Jimba

**Affiliations:** 1 Department of Community and Global Health, Graduate School of Medicine, The University of Tokyo, Tokyo 113-0033, Japan; hodakos@m.u-tokyo.ac.jp (H.K.); shibanuma@m.u-tokyo.ac.jp (A.S.); jkiriya@m.u-tokyo.ac.jp (J.K.); kenicong@m.u-tokyo.ac.jp (K.I.C.O.); 2 UNICEF Uganda Country Office, Kampala P.O. Box 7047, Uganda; smucunguzi@yahoo.ca; 3 Department of Internal Medicine, Mbarara University of Science and Technology, Mbarara P.O. Box 1410, Uganda; conradmuzoora@must.ac.ug

**Keywords:** positive deviance, dual-method use, contraception, unintended pregnancy, sexually transmitted infection, HIV/AIDS

## Abstract

Dual-method use is the most reliable form of protection against unintended pregnancies and human immunodeficiency virus/sexually transmitted infections (HIV/STIs). Although dual-method use remains uncommon among women in stable relationships, some women do practice it. In this study, we explored the barriers that make dual-method use rare and the behaviors of women who practice dual-method use using a positive deviance framework in Uganda. We screened 150 women using highly effective contraceptives at five health facilities. We identified nine women who practiced dual-method use and 141 women who did not. In a qualitative study, we conducted in-depth interviews with all nine women practicing dual-method use and 10 women randomly selected out of the 141 who did not. We performed a thematic analysis using the positive deviance framework. Regardless of practicing dual-method use or not, women faced perceived barriers against dual-method use, such as partner’s objection, distrust, shyness about introducing condoms into marital relationships, and limited access to condoms. However, women practicing dual-method use had higher levels of risk perception about unintended pregnancies and HIV/STIs. They also engaged in unique behaviors, such as influencing their partners’ condom use by initiating discussions, educating their partners on sexual risks and condom use, and obtaining condoms by themselves. These findings will be useful in developing effective community-led and peer-based interventions promoting dual-method use to reduce the dual burden of unintended pregnancies and HIV/STIs among women in Uganda.

## 1. Introduction

Women of reproductive age bear the dual risk of unintended pregnancies and sexually transmitted infections (STIs), including human immunodeficiency virus (HIV) [1,2]. It is estimated that 44% of pregnancies are unintended [2], while 18.2 million women live with HIV worldwide [3]. In sub-Saharan Africa (SSA), the prevalence of unintended pregnancies is estimated at 29%, ranging from 11% in Nigeria to 55% in Namibia [4]. Women account for 59% of the 980,000 new HIV infections that occur among adults in SSA every year [3]. The majority of HIV transmissions in SSA occur via heterosexual sex [5]. The significant gender disparity in HIV infection in SSA starts when women reach reproductive age. Overall, women in SSA get infected with HIV at least five to seven years earlier in their lives than men do [5].

Highly effective contraceptives (HECs) have been introduced to family planning services to prevent unintended pregnancies in SSA countries during the past decades [6]. HECs include hormonal contraceptives such as injectables, implants, and oral contraceptive pills (OCPs), nonhormonal intrauterine devices (IUDs), and female and male sterilization procedures [7]. These are effective contraceptive methods but cannot prevent HIV and other STIs [8]. Therefore, it is crucial for sexually active women to protect themselves from infections, regardless of whether they are using HECs [9]. 

Dual-method use is the application of an HEC together with a barrier method such as male or female condoms [10]. This practice provides simultaneous protection against unintended pregnancies and HIV/STIs [10]. Although condoms are effective in preventing HIV/STIs, condom use alone is only 85% effective in preventing pregnancies, mainly due to incorrect and inconsistent use [11]. Therefore, dual-method use is considered the most effective form of protection against unintended pregnancies and HIV/STIs [8,12]. Nevertheless, dual-method use is not common in SSA [12]. In Zimbabwe, only 3.8% of married women practiced it with their partners [13]. Most of the relevant research in SSA has focused on dual-method use among women already living with HIV and adolescents. For instance, 39% of women living with HIV and 7% of adolescents aged 15–24 years reported dual-method use in Kenya and South Africa, respectively [14,15]. Among the general population, however, little is known about what proportion of women practice dual-method use.

Condom use is essential to prevent unintended pregnancies and HIV/STIs but is not often practiced in long-term relationships such as marriage [13]. Women are likely to prioritize HECs over condoms as they progress in their relationships with their partners [16,17]. It is often considered inappropriate to use condoms with an intimate partner, especially when women are using HECs, as it interferes with intimacy [13,16]. A trade-off between HECs and condom use has been reported in several countries, regardless of HIV/STI prevalence; the idea that HECs are a replacement for condoms is a barrier limiting the popularity of dual-method use [16,17,18]. When women are using HECs, condom use might be perceived as offering protection against HIV/STIs only; in this situation, insisting on condom use can be interpreted as suspecting one’s partner of infidelity and can become awkward or unacceptable in long-term relationships [17]. However, marital sexual intercourse is a major route of HIV transmission in SSA due to inconsistent and incorrect condom use and extramarital sexual relationships [19,20,21]. The necessity of condom use to prevent HIV/STIs is well understood, but it is often not practiced, especially in stable relationships where there is pressure to express confidence in one’s fidelity to the partner [22]. Several interventions have been introduced to promote dual-method use, yet few have yielded a significant increase in dual-method use [12,23]. 

This research adopted a positive deviance framework to examine women practicing dual-method use with their intimate partners in Uganda. The positive deviance approach is used to discover unique behaviors or strategies practiced, either intentionally or unintentionally, by a subgroup of people who remain free from negative outcomes when many of their peers are not able to do so [24,25]. This approach aims to identify these behaviors in order to disseminate them to the rest of the community through community-led and peer-based interventions [24,25]. The positive deviance approach has addressed complex development challenges that are often difficult for outside experts to address, such as the gender-related and sociocultural barriers that have preserved female genital mutilation in Egypt [24]. The barriers to dual-method use in marriage are likewise complex and may be difficult for health experts outside the community to grasp as a whole picture [13]. Given the limited impact of previous interventions [12], the positive deviance approach could be an ideal means of promoting dual-method use among women in long-term relationships. 

To date, little research has been done using the positive deviance approach to promote dual-method use. This study explored the barriers to dual-method use perceived by women using HECs in stable relationships. This study then used the positive deviance approach to identify unique behaviors associated with dual-method use among positive deviants (PDs; i.e., women using the dual method) within the community, to overcome the common barriers to dual-method use in a semi-urban area in Uganda, where women are at considerable risk of unintended pregnancies and HIV/STIs.

## 2. Methods

### 2.1. Project Overview and Study Setting 

We conducted a qualitative study as Phase I of a larger study to explore dual-method use among women using HECs in stable relationships in the Mbarara District in Southwestern Uganda. It will be followed by Phase II, which is a clustered randomized controlled trial (C-RCT) to test the effectiveness of a positive deviance intervention. We developed the intervention to promote dual-method use among women using HECs based on the findings of the current study [9]. The intervention consists of a combination of clinic- and phone-based counselling and a participatory workshop which are delivered by PDs.

We purposively selected Mbarara District as it represents semi-urban areas with high HIV prevalence among women in the country. An estimated 6.2% of adults aged 15–64 years are HIV-positive in Uganda [26]. The rate is higher among women (7.6%) than among men (4.7%) [26]. HIV prevalence is geographically diverse, ranging from 3.1% in the West Nile to 8.0% in the Central 1 region [26]. The southwestern region has the second-highest HIV prevalence (7.9%), with 9.3% for adult women and 6.3% for adult men [26]. In Uganda, 32% of married women use HECs [27]. Only 2% of married women use condoms, whereas 14% of unmarried women use condoms as a main contraceptive method [27]. Female condoms are also available but remain uncommon among both married and unmarried women [27]. Family planning and male condoms are provided free of charge at all health centers by the Ministry of Health and nongovernmental organizations. Condoms are also available for purchase at markets and pharmacies [28].

### 2.2. Study Participants

We screened 150 women to identify women practicing dual-method use. The inclusion criteria for the screening were (i) 18–49 years old, (ii) sexually active, (iii) using HECs, (iv) have a desire to avoid pregnancy for 12 months from recruitment, (v) have a husband or live-in sexual partner, and (vi) have access to a valid phone number. We defined being sexually active as having had sexual intercourse in the three months prior to the screening [13]. We excluded pregnant women and women who were infertile for other reasons, healthcare workers, political and religious leaders, and teachers, because they might not be representative of their communities as they could be influenced by their occupation and social status. The sample size was calculated based on the assumption that at least 7% of women would practice dual-method use and approximately 10 women considered PDs would be identified and invited for an in-depth interview [9]. We also randomly invited 10 women out of those who were not practicing dual-method use, or non-PDs, to an in-depth interview for comparison. The sample size of non-PDs joining the interview was decided to correspond with the expected number of PDs to be identified. 

### 2.3. Participant Enrolment

We approached women at five health facilities to recruit 150 participants eligible for the screening. We purposively selected the five health facilities for their urban or rural status and their size. We selected two county-level health centers (Health Center IV) and three sub-county-level health centers (Health Center III) which have a relatively large number of patients. Two facilities are located in urban areas, and the other three are located in rural areas.

Female research assistants approached women who visited the family planning sections of the selected health facilities for family planning counseling, renewing HECs, or any other purposes. After the first woman had been selected purposively, every third woman was informed about the opportunity to participate in the screening. When a woman was interested in participating, the research assistants confirmed her HEC use with her family planning client record card and asked questions to check her eligibility. This process was repeated until the required sample size was met. In total, 288 women were asked eligibility questions. All women were given an explanation of the purpose of the study and its ethical considerations before they answered the questions.

In the screening process, we conducted face-to-face interviews to collect data on sociodemographic characteristics such as age, education, religion, and the availability of 18 household assets; type of HECs in use; frequency of condom use in the past two months; and history of unintended pregnancy and HIV/STIs, using a structured questionnaire either in English (Table S1) or Runyankore (Table S2). We assessed the frequency of condom use with the item, “How often did you and your partner use a male or female condom during the past two months?” and the answers were reported on a four-point scale: “every time,” “almost every time,” “sometimes,” and “never.” Then, we assessed HEC use with the item, “Apart from condoms, have you been using any other forms of protection against pregnancy during the past two months?” If the answer to this question was “yes,” we asked the women which methods they had been using. We considered only those who used condoms every time along with HECs as PDs practicing dual-method use.

We invited all PDs and 10 non-PDs for an interview on another day. We stratified non-PDs based on the facilities at which they were recruited and then randomly selected two from each facility based on the list of participants who were identified as non-PDs, using computer-generated random numbers. 

### 2.4. Data Collection 

Trained female research assistants conducted in-depth interviews in either English or Runyankore at the selected health facilities in October 2019. Our in-depth interviews focused on the following domains: (1) perceptions of condom use and contraception, (2) reasons and motivations for condom use or nonuse, (3) negotiation and communication regarding condom use, (4) risk perceptions of unintended pregnancy and HIV/STIs, and (5) knowledge of dual-method use. We concluded interviews when data saturation was reached. Each interview lasted about 45–60 min. The interview guide was adapted and modified from a validated in-depth interview tool [29] and is available as a supplementary file (Table S3). We recorded each interview with participants’ consent. We provided all those who participated in the interviews with 10,000 Ugandan shillings (UGX) (equivalent to 3 USD) for their time and transportation. 

### 2.5. Data Analysis

We summarized the participants’ basic characteristics using descriptive statistics. We then conducted the thematic analysis using the positive deviance framework [24,25]. We applied this framework to identify uncommon behaviors of women practicing dual-method use compared to their peers and how such behaviors could facilitate dual-method use by addressing the identified barriers [24,25]. Research assistants transcribed the audio-recorded interview data verbatim, and if they were not in English, translated them from Runyankore into English. The researcher (SM) compared translated transcriptions with recorded data to ensure their accuracy. Two authors (HK and SM) then read all transcripts to identify overarching themes related to barriers to dual-method use and unique behaviors of PDs that can address these barriers and coded areas of the transcripts related to each theme independently using MAXQDA, version 18. The two authors validated identified themes and codes through continuous dialogue until they reached consensus. We used the Consolidated Criteria for Reporting Qualitative Studies (COREQ) guidelines in the reporting of this study (Table S4) [30].

### 2.6. Ethics

We obtained ethical approval from the Research Ethics Committee of the Graduate School of Medicine, the University of Tokyo, Japan (2019085NI); the Institutional Research and Ethics Committee of Mbarara University of Science and Technology, Uganda (IRB15/06-19); and Uganda National Council of Science and Technology (UNCST), Uganda (HS439ES). Participation in this study was voluntary. Research assistants explained the study objective, protocol, and ethical considerations verbally based on an information sheet. The participants were allowed to ask questions about the study before deciding to participate. The research assistants read the questionnaire out aloud to all the participants, as some were expected to be illiterate. About 10% of the 150 participants were illiterate. We conducted each interview in a confidential and secure environment. All the research assistants received a two-day training session on data collection and ethical considerations before the data collection.

## 3. Results

### 3.1. Participant Characteristics

Among the 150 women, nine (6%) were identified as PDs. These nine PDs and 10 non-PDs attended the interviews. Table 1 presents the characteristics of the 19 women who participated in the interviews. Their mean age was 27.7 (standard deviation [SD] 5.9) years. Of these 19 women, 16 (84.3%) had completed primary education or more. The majority (79%) were Roman Catholic, Protestant, or other Christians. All women had given birth at least once, and the mean number of children was 2.5 (SD 1.8). The majority of the women (73.7%) used injectables, and 21.1% used implants. Only one woman used OCPs. The detailed characteristics of participants are presented as a supplementary file (Table S5).

The themes that emerged from the interviews are presented in the following sections. We identified eight main themes, including four barriers to dual-method use and four positive deviant behaviors of women practicing dual-method use. The first section represents common barriers to practicing dual-method use, and the second section describes the unique behaviors and strategies practiced by PDs. 

### 3.2. Barriers to Dual-Method Use for Women Using HECs in Stable Relationships 

Both PDs and non-PDs faced several challenges, including a low level of perceived risk of unintended pregnancies and HIV/STIs. Table 2 outlines all the barriers to dual-method use that emerged from the interviews with PDs and non-PDs. Only barriers associated with HEC use are presented in this section. Other barriers observed in the interviews are presented in a supplementary file (Text S6). Throughout the results section, quotes from the participants are presented to illustrate the findings. 

#### 3.2.1. Low Perceived Need for Dual-Method Use

Regarding condom use, all women who participated in the study understood that condoms are necessary to prevent HIV/STIs. However, six non-PDs did not perceive the need to use condoms with their partners while also using HECs for several reasons. The most common reason (n = 6) was that they believed that their partners were reliably faithful such that there was no risk for him to get infected with HIV and hence, no need to use condoms while also using HECs. Four non-PDs had stopped using condoms with their partners after they had started using HECs. One of them explained,

“I trust my husband, and he trusts me as well. We have always lived together for a long time. Why do we need to use a condom? I am using injection now, and I am no longer worried about being pregnant again.”

(Non-PD, 36 years old, 1-025)

#### 3.2.2. Difficulty Suggesting and Persuading Partners to Use Condoms

Even women who were aware of the risk of HIV/STIs from their partners found it difficult to discuss condom use due to fears of objection and rejection by their partners. Two non-PDs had never discussed condom use with their partners, although they felt that they were at risk of STIs and had used condoms before. Seven PDs and three non-PDs had experienced objection from their partners. The three non-PDs had been discouraged from suggesting condom use again after meeting with opposition from their partners. A non-PD talked about her experience trying to persuade her partner and how he disagreed on the grounds of her HEC use and trust in their marital relationship.

“My husband often travels for his work, so I do not know if he has slept with other women or not. I suggested that he use a condom. But he did not agree because I was using family planning. He said we should trust each other. I could not say anything. I am not sure if it is safe to sleep with him until I know the HIV result.”

(Non-PD, 24 years old, 5-017)

### 3.3. Positive Deviant Behavior of Women Practicing Dual-Method Use in Stable Relationships

PDs reported several behaviors that could enable them to tackle the existing barriers to dual-method use and that non-PDs were not practicing (Table 3). Two common patterns emerged during the analyses: either (1) their partners had supported or suggested dual-method use from the beginning, or (2) their partners had initially had a negative attitude about it, and the women had to persuade them. The former pattern was represented by only two PDs (1-022, 5-004). All of the PDs had practiced dual-method use consistently for at least the past two months, regardless of their partners’ initial reaction to the suggestion of dual-method use.

#### 3.3.1. High Perceived Risk of Unintended Pregnancies and HIV/STIs

Individual risk perception of unintended pregnancy and HIV/STIs can be a factor underlying the unique behaviors of PDs. All PDs, unlike many non-PDs, demonstrated higher levels of perceived threat related to the dual risk. Like non-PDs, the majority of PDs knew their partner’s HIV status. Nevertheless, they expressed their concerns about the risk of HIV/STIs and unintended pregnancy.

Five PDs used condoms in addition to an HEC as a means of mitigating the risk of HIV/STIs. All of them reported that they thought condom use was necessary regardless of HEC use. One PD described her high level of risk perception about STIs:

“My partner and I have been married for years, but I understand all human beings can make mistakes, especially men these days. I am not saying my husband is not trustworthy, but using condoms gives me a sense of relief when I have sexual intercourse with him.”

(PD, 31 years old, 3-024)

Another PD initiated condom use with her partner after experiencing an STI.

“I got an STI shortly after my husband came back from his travel. I knew it came from him. I thought it was not only one time but would happen again. So, I decided to use condoms and discussed with him. That is how I started using condoms in our relationship.”

(PD, 25 years old, 2-014)

Another PD said that she was using condoms as a backup method to prevent unintended pregnancies in case her primary method fails. She mentioned that HECs do not give 100% protection against pregnancies.

“I have been using injectables, but I sometimes forget when it will be expired. To prevent pregnancy in such cases, I believe it is important to use both. I know some friends of mine have got pregnant somehow while using family planning methods. I do not know if it is true, but I heard injectables can expire before three months because they receive an insufficient dose. So, I believe condoms are necessary for women who want to avoid pregnancy.”

(PD, 18 years old, 2-002)

Three other PDs mentioned that they were practicing dual-method use to prevent both pregnancies and HIV/STIs, as HECs and condoms can work as backup protection for one another. One PD described her perceived need for dual-method use:

“Condoms can prevent me from getting pregnant and infections from my partner. But I have experienced condoms slipping several times. I already have two children, so we do not want to have any more babies. That is why using two different methods is important to me.”

(PD, 22 years old, 5-023)

#### 3.3.2. Effective Communication about Dual-Method Use

Notably, PDs had taken several steps to initiate and continue discussions about dual-method use even though their partners had initially expressed a negative attitude toward it. Many of them regularly discussed condom use with their partners before sexual intercourse.

- Initiating discussions of condom use

All PDs had initiated discussions of condom use with their partners. To facilitate their initial discussions, they had used a unique strategy of sharing health education messages that they had received with their partners. All non-PDs and PDs mentioned that they received such messages about family planning and HIV/STI prevention at some point in the past when they visited health facilities. Five PDs then shared the messages with their partners to start a discussion upon returning home. Some of them mentioned that it seemed easier to bring up a sensitive topic such as condom use and to persuade their partners by sharing what they had learned rather than bringing the subject up without context.

“I often visited the health facility for postnatal care and family planning after delivering my child. I was given counseling on family planning and condom use. I got worried about HIV infection and told my partner about what I learned one day. He also got interested and started discussing condom use.”

(PD, 27 years old, 2-004).

- Repeatedly suggesting condom use 

Almost all PDs, except for two whose partners had not expressed opposition during the first discussion, repeatedly suggested using condoms even after their partners had responded negatively. Three PDs reported that they had avoided having sexual intercourse without a condom, sometimes even for a few months, until their husbands had agreed to use condoms.

“I do not trust my partner as he often travels for work, and he avoids testing for HIV together. He did not agree to use condoms when I suggested it, so I avoided having sex with him several times, maybe for three months. He was not happy, but I thought it was important to protect myself.”

(PD, 27 years old, 2-004).

The same PD also mentioned that, although her partner was not happy, he had never been violent toward her. To avoid conflicts, some PDs said that they found it was crucial to discuss condom use with their partners in a gentle manner, often long before sexual intercourse as opposed to during an intimate moment. For instance, the same PD quoted above reported that she had kept bringing up the subject of their HIV/STI risk and condom use when her husband came home from his travels.

“If I tell him to use a condom suddenly before having sex, he may get surprised and angry, thinking I am accusing him of infidelity. If he gets mad and tries to defend himself, it is difficult to keep discussing it. So, I brought up this sensitive topic when he first came back home from his travels, when he seemed to be in a good mood, in the same way that we talk about ordinary things.”

(PD, 27 years old, 2-004).

Another PD reported that she had persuaded her partner without causing tension in their relationship with her unique strategy.

“I always talked with him in a gentle manner, not accusing him of his sexual behavior. I talked about HIV and STI risk without condom use in a general setting, not between him and me, like a teacher. He likes discussions and tries to show off his knowledge. I think it helped him realize he did not use condoms although he explained the importance of condom use.”

(PD, 31 years old, 3-024)

- Referring to unintended pregnancies and STI incidence in the past

Three PDs suggested condom use to their partners by referring to their own experiences with unintended pregnancies and STIs in the past. One of them mentioned that she had often told her partner how shocked she was to get an STI in the past as a means of facilitating discussion with him without conflict.

“I did not hide from him that I had got an infection before, which I might have got from my partner. I always mentioned how sad I was at that time when I suggested using condoms. But I did not accuse him of this, because it creates tension between us. I always tried to be calm when talking about condom use with him. Now he understands I am really serious about STIs and always uses condoms without me reminding him to do so.”

(PD, 27 years old, 4-010)

- Emphasizing the family planning aspect of condoms over their role in HIV/STI protection 

One PD had intentionally hidden that she was using an HEC from her partner. This strategy might have helped her avoid the issue of distrust while persuading her husband to use condoms by emphasizing the family planning aspect of condoms over their HIV/STI protection aspect.

“I did not tell him that I was using family planning. I had been using it before we got married, but I had never told him. I knew he would not agree to use condoms if he knew that I would not get pregnant because of injections.”

(PD, 27 years old, 4-010).

- Avoiding having sex after drinking alcohol

Two PDs said that they tried to avoid having sexual intercourse when their husbands were drunk. This might have enabled them to avoid incorrect condom use as well as disagreements over condom use that could be more likely to occur when one of the partners has been drinking.

“I avoid sex when my husband has been drinking. It is hard to discuss condom use rationally with him when he is drunk, and we may end up arguing. I just leave him until he falls asleep by himself.”

(PD, 31 years old, 3-024).

#### 3.3.3. Instructing Their Partners on How to Use A Condom

Condom use is often considered a male responsibility [31]. However, two of the PDs in this study had not only suggested condom use but also instructed their partners on how to use condoms. The women had learned how to use condoms correctly through various means, including family planning counseling, postnatal visits, and workshops hosted by NGOs.

“I learned how to put on a male condom during family planning counseling. I even practiced with a model penis at that time. I returned home with a few condoms and showed him how it works. We had never used condoms before, but it went smoothly. I am sure I am handier than him.”

(PD, 25 years old, 2-014)

“I taught him the right way to use condoms. It will help him avoid getting HIV and other STIs even if he is sleeping with another woman while he is away. This means that it can protect me and my children as well in the end.”

(PD, 40 years old, 1-022)

#### 3.3.4. Obtaining Condoms from A Variety of Sources 

Some PDs obtained condoms from several sources. This practice preserved their access to condoms even if one source failed. One PD said that, although her partner would not agree to use the condoms provided at health facilities, he did not object to using condoms bought at the market.

“My partner refused to use condoms when I suggested it, saying they smelled bad. I, actually, also hate the rubber smell of free condoms. So, I bought condoms from a pharmacy. They have nice smells such as chocolate and vanilla, and he said he wanted to try them. I think there are many types, so if people do not like the free condoms, they might consider trying purchased ones.”

(PD, 21 years old, 5-021)

Notably, the majority of PDs took charge of obtaining the condoms they needed rather than relying on their male partners to do so, though many non-PD women relied on condoms provided by their partners and felt uncomfortable being seen buying condoms in a public place. One PD explained how she had taken control of condom use in her relationship by buying them herself and keeping them in convenient places such as her purse and bedroom.

“It is difficult for him to buy condoms, so I receive condoms when I visit the health facility. I am not afraid because I am the one who is at risk, and it is my responsibility to protect myself. I keep them in my purse [showing condoms to the interviewer].”

(PD, 40 years old, 1-022)

Another PD mentioned that she received condoms from nurses when she visited health centers if the dispenser was out of stock.

“When I cannot get condoms from the dispenser at the health center, I normally talk to the nurses because they have some stock and are willing to give them to patients.”

(PD, 22 years old, 5-023)

Some PDs said that, although they feel embarrassed about being seen receiving condoms from a health facility, they manage to access condoms consistently. One of them visited a health facility when there were fewer patients around.

“I feel embarrassed[receiving condoms in front of many people, but I can take condoms without feeling any shyness by going there when there are not so many people, like late afternoon and evening.”

(PD, 31 years old, 3-024)

## 4. Discussion

The majority of both PDs and non-PDs faced several perceived barriers to using the dual method, such as partner’s objection, distrust, and shyness about introducing condoms into marital relationships, as well as limited access to and poor quality of condoms. The PDs overcame these barriers through unique behaviors and strategies by exerting significant control over condom use in their relationships by initiating discussions, educating their partners on sexual risks and condom use, and accessing condoms. 

The majority of non-PDs did not perceive the need for dual-method use, as they trusted in their partners’ marital fidelity and were using HECs to prevent unintended pregnancies. HEC use can decrease women’s perceived risk of unintended pregnancies and diminish the perceived benefits of condom use as a contraceptive method, especially in marital relationships, which are often associated with intimacy and trust [16]. Although they were aware of the risk of unintended pregnancies and HIV/STIs, two non-PDs had never suggested dual-method use to their partners because they perceived several barriers such as partner’s objection, distrust, and shyness about introducing condoms into marital relationships. Moreover, three other non-PDs had stopped suggesting condom use due to objection from their partners. The effect of marriage on views about condom use has been well studied in diverse sociocultural contexts. In Malawi, for instance, the perceived acceptability of condom use decreased among couples after they got married [21]. Another study reported the psychological barriers that women experience with regard to introducing condom use in marital sexual intercourse and the negative attitudes among both men and women toward condom use in rural Kenya [32]. Furthermore, HEC use can increase perceived barriers to using condoms with marital partners as it provides protection against pregnancy. HEC users reported more inconsistent condom use than non-HEC users in Kenya, Brazil, and the United States of America [16,17,18]. Because of these perceived barriers, condom use may remain low among married couples. 

Besides these perceived barriers, women in this study faced other barriers such as limited access to and poor quality of condoms. Few women reported that the financial cost of condoms was a barrier to dual-method use; this is in line with other studies in SSA [33,34]. Additionally, some PDs and non-PDs felt embarrassed being seen receiving condoms in a public place such as a health facility. In addition, a few PDs and non-PDs reported inadequate supplies of condoms at health facilities. Some PDs and non-PDs mentioned they did not like the rubber-and-lubricant smell of the free condoms and the itchiness that some condoms can cause. All of these aspects have previously been reported as issues limiting condom use in SSA [34] and are barriers to dual-method use. 

Many PDs, however, overcame the perceived barriers to introducing condoms into marital relationships through effective communication, even though their partners had initially objected. Five PDs reported that they had received health education messages and then shared these with their partners as a means of initiating a discussion about condom use. In a qualitative study of married couples using condoms in Uganda, the majority of couples agreed that the female partner had first suggested condom use [19]. Presumably, women receive more messages about family planning and condom use at health facilities than men do, and sharing such messages makes it easier for them to start talking about condom use. Moreover, PDs continued to try to persuade their partners to use condoms even after initial rejection. Three PDs refused to engage in sexual intercourse without a condom when their partners did not agree. Married women in Uganda have used this strategy to make their partners understand that they are serious about condom use [19]. One PD intentionally hid the fact that she was using an HEC to make her husband believe that condoms were necessary to prevent unintended pregnancy. Women can avoid implying that they distrust their husbands’ fidelity by emphasizing the family planning aspect of condoms over their role in protection against HIV/STIs [19,31]. To avoid arguing with their partners, some PDs first suggested condom use calmly when their partners were in a good mood and well in advance of sexual intercourse. Two PDs not only recommended condom use but also instructed their partners on how to use condoms correctly.

Moreover, the PDs managed to access condoms consistently. Whereas other women did not obtain condoms because of their shyness and instead relied on their partners to provide condoms, many PDs had several sources for condoms and obtained and stored condoms on their own. One PD received condoms from nurses when she found the dispenser empty; this might be an option for other women who feel embarrassed to receive condoms in front of other people. Another PD convinced her partner to view condom use more favorably and persuaded him to use them by buying higher-quality flavored condoms from pharmacies. 

Some of these strategies are not necessarily unique or uncommon means of promoting dual-method use and have been well studied in the context of condom use. It is likely, however, that each of them helped PDs in this study to practice dual-method use, which is uncommon among married women even in high-HIV prevalence settings such as SSA. Some barriers, such as the inadequate supply and poor quality of condoms, are not solvable through the positive deviance approach, as this approach focuses on behavioral changes [35]. However, some of these technical issues can be sidestepped through behavioral changes. For instance, introducing a variety of sources for condoms can help women consistently access condoms even if one source runs out [13].

This study has some limitations. First, our findings must be interpreted with caution, as they are the result of a small number of interviews. Our sample size of PDs (n = 9) might not be large enough to provide a full picture of their unique behaviors and strategies [36]. Second, this study is cross-sectional and, therefore, cannot be used to establish a cause-and-effect relationship between observed behaviors and dual-method use. Results could not be attributed to the PDs alone because PDs might have been exposed to other interventions and external factors such as gender power balance [37]. Moreover, this study did not fully explore why PDs had a higher level of perceived threat for the dual risk, which might be a factor underlying their unique behaviors. In Phase II of our study, we will examine the effectiveness of an intervention that we are developing based on the unique practices found in the interviews through a randomized controlled trial with a larger sample of women using HECs [9]. Finally, this study focused exclusively on women, although condom use is not an individual action that a woman alone can choose to undertake [32]. Their partners’ attitudes and experiences certainly influenced their willingness to practice dual-method use, but the present study could not explore any such factors related to the male partners. As such, further study is recommended. Nevertheless, to our knowledge, this is the first study to explore the unique behaviors of women practicing dual-method use in stable relationships with the positive deviance approach. The findings of this study will be useful to public health policymakers in developing programs to reach women who need dual-method use, which will reduce unintended pregnancies and HIV/STIs. 

Based on these findings, future interventions should consider the following points. First, it is critical to raise awareness among women using HECs that they are at risk of getting HIV/STIs from their marital partners and that dual-method use is the most reliable method of preventing unintended pregnancies and HIV/STIs [8]. We propose using the real voices of PDs as peer educators who can share their experiences to encourage their peers to discuss and define their own risk and identify the best solutions to prevent HIV/STIs. Second, it could be useful to provide women using HECs with health education about condom use in the form of handouts in the local language and to encourage them to share the topic with their partners. For PDs, informational messages received from health facilities served as conversation starters, enabling them to discuss HIV/STI risk and condom use while reducing the feeling of shyness and awkwardness in bringing up these sensitive topics. The results show that even health messages that are delivered to women only can influence condom use. Third, it might be useful to engage in role-playing to help women decide how to start and continue discussions about condom use without arguing with their male partners. Role-play enables participants to take on different roles, share common reactions of their male partners when they suggest condom use, and examine issues in communication together with their peers. PDs can provide feedback based on their own experiences. This could contribute to reducing fear of partner’s objection and shyness about suggesting condom use among participants and trigger their actions in a real setting. Fourth, it might be helpful for some women to have more information on the variety of sources for condoms and the types of condoms available at pharmacies and markets, as some men and women dislike the free condoms. Lastly, it will be helpful to provide instructions on how to use condoms, as well as practice sessions for women so that they can help their partners use condoms correctly.

Based on these findings and above-mentioned plans, we developed an intervention for the Phase II of the whole study to promote dual-method use among women using HECs through a C-RCT. The intervention consists of a combination of clinic- and phone-based counselling and a participatory workshop delivered by PDs to disseminate their effective behaviors for dual-method use. 

## 5. Conclusions

This study focused on women practicing dual-method use in stable relationships, a rare combination, using the positive deviance approach. The majority of women in this study faced several perceived barriers such as partner’s objection, distrust, and shyness about introducing condoms into marital relationships, as well as limited access to and poor quality of condoms. However, the PDs significantly influenced condom use in their relationships by initiating discussions, educating their partners on sexual risks and condom use, and obtaining condoms, in contrast to their non-PD peers. Given that the majority of HIV/STIs in SSA occur among married couples, it is critical for women who are at risk to protect themselves with dual-method use. Therefore, these findings underscore the importance of warning the public about the risk of unprotected sexual intercourse among married couples and developing effective interventions to promote dual-method use among women using HECs. Women using HECs can be more accessible than men, as they visit family planning clinics to obtain their HECs; as a result, interventions aimed at women are expected to be more cost-effective in preventing the dual risk of unintended pregnancies and HIV/STIs in Uganda.

## Figures and Tables

**Table 1:** Characteristics of participants (n = 19).

Variables	PDs (n = 9)		Non-PD (n = 10)		Total (n = 19)	
	n	%	n	%	n	%
Age, mean in years (SD)	25.9	(6.6)	29.3	(5.0)	27.7	(5.9)
Education						
None	2	22.2	1	10.0	3	15.8
Primary	6	66.7	9	90.0	15	79.0
Secondary or more	1	11.1	0	0.0	1	5.3
Religion						
Roman Catholic	3	33.3	2	20.0	5	26.3
Protestant/Other Christian	3	33.3	7	70.0	10	52.6
Muslim	3	33.3	1	10.0	4	21.1
Ever given birth						
No	0	0.0	0	0.0	0	0.0
Yes	9	100.0	10	100.0	19	100.0
No. of children, mean (SD)	2.4	(2.2)	2.6	(1.4)	2.5	(1.8)
Type of HECs						
Injectables	7	77.8	7	70.0	14	73.7
Implants	1	11.1	3	30.0	4	21.1
OCPs	1	11.1	0	0.0	1	5.3

PDs: positive deviants; SD: standard deviation; HECs: highly effective contraceptives; OCPs: oral contraceptive pills.

**Table 2:** Barriers to dual-method use for women using highly effective contraceptives (HECs) in stable relationships.

Theme	Description
Low perceived need for dual-method use	Women had low perceived need of using condoms with their partners due to - Trust - HIV-negative status - HEC use
Difficulty suggesting and persuading partners to use condoms when women are aware of the risk of HIV/STIs from their partners	Women had never discussed dual-method use with their partners due to - Fear of rejection - Fear of causing distrust with partners - Feeling awkward about introducing condoms into a long-term relationship Women had stopped discussing condom use after partner objection due to - Trust - HEC use
Limited access to condoms	Women had limited access to a condom when necessary due to - Financial burden - Using only condoms given by partners - Feeling shy about obtaining condoms - Supply shortages
Concerns about condom use	Women had concerns about condom use, such as - Bad smells - Itchiness and rashes - Breakage of condoms - Myth that condoms cause cancers - Reduction in sexual urge and satisfaction

**Table 3:** Positive deviant behaviors of women practicing dual-method use in stable relationships.

Theme	Description
High perceived risk of unintended pregnancies and HIV/STIs	Women had high levels of risk perception of unintended pregnancy and HIV/STIs regardless of HEC use
Effective communication about dual-method use	Women communicated with partners to practice dual-method use effectively through - Initiating discussions about condom use by forwarding health education messages to their partners - Repeatedly suggesting condom use by refusing to have sexual intercourse without a condom and continuing to talk in a gentle manner prior to sexual intercourse - Referring to unintended pregnancies and STI incidence in the past - Emphasizing the family planning aspect of condoms over STI protection - Avoiding sexual intercourse after drinking alcohol
Instructing partners on how to use a condom	Women instructed their partners on how to use condoms
Obtaining condoms from a variety of sources	Women obtained condoms from several sources such as health facilities and pharmacies to preserve their access to a variety of condoms

## Supplementary Materials

The following are available online at https://www.mdpi.com/1660-4601/17/14/5009/s1, Table S1: questionnaire (English); Table S2: questionnaire (Runyankore); Table S3: interview guide; Table S4: consolidated criteria for reporting qualitative studies (COREQ): a 32-item checklist; Table S5: the detailed characteristics of participants included in in-depth interviews; Text S6: barriers to dual-method use which were not directly related to HEC use for women in stable relationships.
