# Supplementary material for: Positive Deviance for Dual-Method Promotion among Women in Uganda: A Qualitative Study
Source: Int J Environ Res Public Health. 2020 Jul 12;17(14):5009. doi: 10.3390/ijerph17145009 (PMC7400262; doi:10.3390/ijerph17145009)
Supplement: Supplementary file 1 [file ijerph-17-05009-s001.zip › Table S1-Questionnaire (English).pdf]

Table S1: Questionnaire (English)

NO. □-□□□

QUESTIONAIRE (Phase I)

Introduction:

"My name is... We are interviewing people in Mbarara district, Uganda about family planning methods and condom use. Have you been interviewed in the past few weeks for this study? "

If the respondent has been interviewed before, do not interview this person again. Tell them you cannot interview them a second time, thank them, and end the interview. If she has not been interviewed before, explain the study using the information sheet.

If the respondent agrees to participate, ask her the following questions.

Screening question:

1. How old are you? *Check yes if she is between 18-49 years old.*	Yes	·	No
2. Do you have a husband or partner living together over 3 months?	Yes	·	No
3. Have you had sexual intercourse with him in the past 3 months?	Yes	·	No
4. Do you want to avoid pregnancies in the next 12 months?	Yes	·	No
5. Are you using any family planning methods such as pills, implants, and injectables?	Yes	·	No
6. Do you have a working telephone number?	Yes	·	No
7. Are you pregnant or infertile?	Yes	·	No
8. Are you either a health worker, political or religious leader, or teacher?	Yes	·	No

If the respondent answers yes to 1-6 and no to 7-8, she is eligible for this study, ask her to give the informed consent and start the interview.

If the respondent has proved not to be eligible for the study, tell her you cannot interview her, thank her, and end the interview.

Identification:

Date	D D / M M / Y Y Y Y
Facility	
Interviewer	
Field editor	

NO. □-□□□

QUESTIONAIRE

Date: D D / M M / Y Y Y Y
Facility:
Interviewer:
Field editor:

A) BACKGROUIND INFORMATION

	Questions	Responses	Skip
A-1	What is your purpose of visiting this health center today?	☐$_0$ To initiate family planning<br>☐$_1$ To continue family planning<br>☐$_2$ To receive family planning counselling<br>☐$_3$ Other ( )	
A-2	How old are you? (Completed year)	Age in completed years............................ □□<br>☐$_8$ Don't know	
A-3	What is your ethnicity?	☐$_1$ Banyankole<br>☐$_2$ Baganda<br>☐$_3$ Bakiga<br>☐$_4$ Batoro<br>☐$_5$ Acholi<br>☐$_6$ Other ( )	
A-4	What is your religion?	☐$_1$ Roman catholic<br>☐$_2$ Protestant/other Christian<br>☐$_3$ Muslim<br>☐$_4$ No religion<br>☐$_5$ Other ( )	
A-5	What is the highest level of school you have attended?	☐$_0$ Never<br>☐$_1$ Incomplete primary school<br>☐$_2$ Complete primary school<br>☐$_3$ Incomplete secondary school<br>☐$_4$ Complete secondary school<br>☐$_5$ Higher than secondary school	
A-6	What is your occupation?	☐$_0$ Unemployed<br>☐$_1$ Agriculture<br>☐$_2$ Government worker<br>☐$_3$ Private sector<br>☐$_4$ Own business<br>☐$_5$ Students<br>☐$_6$ Other ( )	
A-7	Does your household have:<br>a) Electricity?<br>b) A radio?<br>c) A television?<br>d) A mobile phone?<br>e) A non-mobile phone?<br>f) A refrigerator?<br>g) A solar panel?<br>h) A table?<br>i) A chair?<br>j) A sofa?<br>k) A bed?<br>l) A cupboard?<br>m) A clock?<br>n) A microwave oven?<br>o) A DVD player?<br>p) A cassette or CD player?<br>q) A car or truck?<br>r) A bicycle?	NO YES<br>a) Electricity ☐$_0$ ☐$_1$<br>b) Radio ☐$_0$ ☐$_1$<br>c) Television ☐$_0$ ☐$_1$<br>d) Mobile phone ☐$_0$ ☐$_1$<br>e) Non-mobile phone ☐$_0$ ☐$_1$<br>f) Refrigerator ☐$_0$ ☐$_1$<br>g) Solar panel ☐$_0$ ☐$_1$<br>h) Table ☐$_0$ ☐$_1$<br>i) Chair ☐$_0$ ☐$_1$<br>j) Sofa ☐$_0$ ☐$_1$<br>k) Bed ☐$_0$ ☐$_1$<br>l) Cupboard ☐$_0$ ☐$_1$<br>m) Clock ☐$_0$ ☐$_1$<br>n) Microwave oven ☐$_0$ ☐$_1$<br>o) DVD player ☐$_0$ ☐$_1$<br>p) Cassette or CD player ☐$_0$ ☐$_1$<br>q) Car or truck ☐$_0$ ☐$_1$<br>r) Bicycle ☐$_0$ ☐$_1$	
A-8	What is your marital status?	☐$_1$ Single<br>☐$_2$ Married<br>☐$_3$ Cohabiting<br>☐$_4$ Widow<br>☐$_5$ Divorced	
A-9	Are you currently married or living with a man with whom you have a sexual relationship?	☐$_1$ Currently married, living with a spouse<br>☐$_2$ Currently married, living with another sexual partner<br>☐$_3$ Currently married, not living with a spouse or any other sexual partners<br>☐$_4$ Not married, living with a sexual partner<br>☐$_5$ Not married, not living with a sexual partner	 <br><br>→A-11<br>→Check Criteria
A-10	Does your husband have other wives?	☐$_0$ No<br>☐$_1$ Yes<br>☐$_8$ Don't know	
A-11	Have you ever given birth?	☐$_0$ No<br>☐$_1$ Yes	→A-15
A-12	Have you ever had an unintended pregnancy?	☐$_0$ No<br>☐$_1$ Yes	
A-13	How many children have you ever delivered?<br>IF NONE, RECORD '00'.	.... □□	
A-14	How many living children do you have?<br>IF NONE, RECORD '00'.	.... □□	
A-15	Would you like to have (a/another) child in the future, or would you prefer not to have any (more) children?	☐$_0$ No more<br>☐$_1$ Have (a/another) child<br>☐$_2$ Undecided	
A-16	Would your husband / live-in sexual partner like to have (a/another) child in the future, or would he prefer not to have any (more) children?	☐$_0$ No more<br>☐$_1$ Have (a/another) child<br>☐$_2$ Undecided<br>☐$_8$ Don't know	

B) CONTRACEPTIVE USE and HIV/STIs

	Questions	Responses	Skip
B-1	Did you use a male or female condom the last time you had sexual relations with your husband or live-in sexual partner?	□0 No<br>□1 Yes, male condom<br>□2 Yes, fe male condom	
B-2	Apart from condoms, have you been using any other forms of protection against pregnancy during the past two months?	□0 No<br>□1 Yes	→Check Criteria
B-3	Which method have you been using?	□1 Injectables<br>□2 Implants<br>□3 IUD<br>□4 Pill<br>□5 Female sterilization<br>□6 Male sterilization<br>□7 Other method ( )	
B-4	Since what month and year have you been using (METHOD ON LIST IN B-3) without stopping?	Month .... □□<br>□88 Don't know month<br>Year .... □□□□<br>□88 Don't know year	
B-5	How often did you and your partner use a male or female condom during the past two months?	□0 Never<br>□1 Sometimes<br>□2 Almost every time<br>□3 Every time<br>□8 Don't know<br>□9 No response	
B-6	Have you ever been told by a health care provider that you had HIV and STIs such as chlamydia, gonorrhea, or trichomonas infections?	□0 No<br>□1 Yes<br>□8 Don't know<br>□9 No response	
