# Supplementary material for: Positive Deviance for Dual-Method Promotion among Women in Uganda: A Qualitative Study
Source: Int J Environ Res Public Health. 2020 Jul 12;17(14):5009. doi: 10.3390/ijerph17145009 (PMC7400262; doi:10.3390/ijerph17145009)
Supplement: Supplementary file 1 [file ijerph-17-05009-s001.zip › Table S2-Questionnaire (Runyankore).pdf]

Table S2: Questionnaire (Runyankore)

NO. □-□□□

QUESTIONAIRE (Phase I)

Introduction:

Amaziina gangye ninye.......... Nitubuuza abatuuragye ba Mbarara district omuri Uganda aha miringo eyo eyokubarira oruzaro hamwe nenkozera y'obupiira. Haine obaire yakuburizeho ebikwatinine nebyo omu sande nkye ezihiwire?

If the respondent has been interviewed before, do not interview this person again. Tell them you cannot interview them a second time, thank them, and end the interview. If she has not been interviewed before, explain the study using the information sheet.

If the respondent agrees to participate, ask her the following questions.

Screening question:

1. Oine emyaka engahi ?gorora ego yaba aine emyaka 18 - 49 y'obukuru	Eego	・	Ngaaha
2. Obwahati oshwirwe ninga shi otwiire n'omushaija owu ori kwegaita nawe omu myezi 3 ehin	Eego	・	Ngaaha
3. Wayegisire nawe omu byomubonano omu myezi 3 ehingwiire	Eego	・	Ngaaha
4.Noyenda kwerinda kutunga enda omu myezi 12 erikwija?	Eego	・	Ngaaha
5.Nokozesa family planning nka obushuma, implants, ninga ebikatu?	Eego	・	Ngaaha
6. Oyine enamber ye ssimu?	Eego	・	Ngaaha
7. Oyine ndaah ninga tokazala?	Eego	・	Ngaaha
8. Nokora omwiraro, omukanisa ninga ori omushomessa?	Eego	・	Ngaaha

If the respondent answers yes to 1-6 and no to 7-8, she is eligible for this study, ask her to give the informed consent and start the interview.

If the respondent has proved not to be eligible for the study, tell her you cannot interview her, thank her, and end the interview.

Identification:

Date	D D / M M / Y Y Y Y
Facility	
Interviewer	
Field editor	

NO. □-□□□

Date:	D D / M M / Y Y Y Y
Facility:	
Interviewer:	
Field editor:	
Phone No 1.	
Phone No 2.	

QUESTIONAIRE

A) BACKGROUND INFORMATION

	Questions (Runyankore)		Responses (Runyankore)	Skip
A-1	Ni nshonga ki eyakureetaha erizooba?	□0<br>□1<br>□2<br>□3	Okutandika family planning<br>Kugumizamu family planning<br>Okutunga obuhabuzi aha bya family planning<br>Endiijo yoona ( )	
A-2	Oyine emyaka engahi?	 □8	Emyaka □□<br>Tindikugimanya	
A-3	Oryomuki?	□1<br>□2<br>□3<br>□4<br>□5<br>□6	Banyankore<br>Baganda<br>Bakiga<br>Batooro<br>Acholi<br>Abandi ( )	
A-4	Noshoma diini ki?	□1<br>□2<br>□3<br>□4<br>□5	Omukatoliki<br>Omukristaayo<br>Omusiraamu<br>Tinyine diini<br>Ediini endiijo	
A-5	Okashoma waahikahi?	□0<br>□1<br>□2<br>□3<br>□4<br>□5	Tinkashomahoga<br>Tindahezize purayimare<br>Nkaheza purayimare<br>Tindaherize siniya<br>Nkaheza siniya<br>Nkarenga aha siniya	
A-6	Nookora murimo ki?	□0<br>□1<br>□2<br>□3<br>□4<br>□5<br>□6	Tinyine murimo<br>Omuhingi / Omuriisa<br>Omukozi wa gavumenti<br>Ninkorera abiine bizineesi<br>Nyine bizineesi yangye<br>Nkiri omwishomero<br>Ekindi kyona ( )	
A-7	Omuk'owaanyu mwine:<br>a) Amashanyarazi?<br>b) Reediyo?<br>c) Tiivi?<br>d) Esimu y'omungaro ?<br>e) Esimu y'omunju?<br>f) Furiigi?<br>g) Amashanyarazi ag'eizooba aga soola?<br>h) Emeeza?<br>i) Entebe?<br>j) Entebe empango za soofa ez'omumuryango?<br>k) Ekitanda?<br>l) Ekabada?<br>m) Eshaaha?<br>n) Akooma kamashanyarazi ak'okutagasizamu?<br>o) Ekyoma ky'okuteera za vidiyo?<br>p) Ekyoma ky'okuteera kaseti ninga CD?<br>q) Emotoka ninga akaroore?<br>r) Egaari?	 a)<br>b)<br>c)<br>d)<br>e)<br>f)<br>g)<br>h)<br>i)<br>j)<br>k)<br>l)<br>m)<br>n)<br>o)<br>p)<br>q)<br>r)	NO YES<br>Amashanyarazi? □0 □1<br>Reediyo? □0 □1<br>Tiivi? □0 □1<br>Esimu y'omungaro □0 □1<br>Esimu y'omunju □0 □1<br>Furiigi □0 □1<br>Amashanyarazi ag'eizooba aga soola? □0 □1<br>Emeeza □0 □1<br>Entebe □0 □1<br>Entebe empango za soofa ez'omumuryango? □0 □1<br>Ekitanda □0 □1<br>Ekabada □0 □1<br>Eshaaha □0 □1<br>Akooma kamashanyarazi ak'okutagasizamu? □0 □1<br>Ekyoma ky'okuteera za vidiyo? □0 □1<br>Ekyoma ky'okuteera kaseti ninga CD? □0 □1<br>Emotoka ninga akaroore □0 □1<br>Egaari □0 □1	
A-8	Waashweire/Waashweirwe?	□1<br>□2<br>□3<br>□4<br>□5	Tikashweirwe<br>Shweirwe<br>Nintuura nomushaija<br>Enfaakazi<br>Tukataana	
A-9	Obwahati oshweirwe nari shi notuura n'omushaija ow'orikwegaita nawe?	□1<br>□2<br>□3<br>□4<br>□5	Nshweirwe, nintuura n'ibanyi<br>Nshweirwe kwonka nintuura n'omushaija ondiijo atari ibanyi<br>Nshweirwe kwonka tinkuntuura n'ibanyi nari shi omushaija ondiijo weena<br>Tinshweirwe kwonka nintuura nomushaija ori nk'ibanyi<br>Tinshweirwe kandi tinkutuura namushaija weena	 <br><br>→A-11<br>→Check Criteria
A-10	Baro nari shi omushaija waawe aine abakazi abandi?	□0<br>□1<br>□8	Ngaaha<br>Eego<br>Tindikumanya	
A-11	Orazaireho??	□0<br>□1	Ngaaha<br>Eego	→A-15
A-12	Oratwaireho enda otakigyendereire?	□0<br>□1	Ngaaha<br>Eego	
A-13	Okazaara abaana bangahi?<br>IF NONE, RECORD '00'.		.... □□	
A-14	Aha baana baawe boona abo wazaire, mbangahi abakiriho?<br>IF NONE, RECORD '00'.		.... □□	
A-15	Noohurira noyenda kuzaarayo ondiijo mwana, ninga aboine nibakumara nari shi tokwenda kuzaara nakakye?	□0<br>□1<br>□2	Tinkyenda ndiijo<br>Ninyenda omwana / Ninyenda omwan'ondiijo<br>Tinkashaziremu	
A-16	Baro, nari shi omushaija ow'orikutuura nawe hati, we akabaire nayenda kuzaarayo ondiijo ninga shi nayenda kuzaara abaana? Nari shi takwenda kuzaara ninga takyenda kuzaara bandi?	□0<br>□1<br>□2<br>□8	Takyenda ndiijo<br>Nayenda omwana / Nayendaa omwan'ondiijo<br>Takashaziremu<br>Tindikumanya	

B) CONTRACEPTIVE USE and HIV/STIs

	Questions (Runtankore)		Responses (Runyankole)	Skip
B-1	Obu wahereruka kwegaita na baro nari shi omushaija waawe okakozesa akapiira? Akapiira kabashaija nari shi akabakazi?	☐0<br>☐1<br>☐2	Ngaaha<br>Eego akapiira akabashaija<br>Eego, akapiira akabakazi	
B-2	Oyihireho obupiira, hariho omuringo ogundi gwona ogw'orikukozesa kureeba ngu tiwagir'enda nari shi tiwatwara?	☐0<br>☐1	Ngaaha<br>Eego	→Check Criteria
B-3	Obwahati nookozesa ki?	☐1<br>☐2<br>☐3<br>☐4<br>☐5<br>☐6<br>☐7	Ekikatu<br>Implants<br>Akaheta<br>Obujuma<br>Okushara enshekye<br>Okushara emisi y'amagosi<br>Omuringo ogundi ( )	
B-4	Okatandika okwezi ki, omwaka ki, okukozesa omuringo ogu waagamba otareekiir'aho?	 ☐88<br><br>☐88	Okwezi.. ☐☐<br>Tinkumanya kwezi<br>Omwaka .... ☐☐☐☐<br>Tinkumanya mwaka	
B-5	Omu myezi ebiri eyenyima okakozesaho akapiira kabakazi nari shi akabashaija?	☐0<br>☐1<br>☐2<br>☐3<br>☐8<br>☐9	Tinkakakozesahoga<br>Obumwe n'obumwe<br>Haihi buri mwanya<br>Buri mwanya<br>Tindinkumanya<br>Tikyagarukwamu	
B-6	Omushaho arakugambiireho ngu oine akakooko ka siliimu nari shi endwara z'obushambani nk'enziku, chlamydia or trichomonas?	☐0<br>☐1<br>☐8<br>☐9	Ngaaha<br>Eego<br>Tindikumanya<br>Tikyagarukwamu	
