# Supplementary material for: Positive Deviance for Dual-Method Promotion among Women in Uganda: A Qualitative Study
Source: Int J Environ Res Public Health. 2020 Jul 12;17(14):5009. doi: 10.3390/ijerph17145009 (PMC7400262; doi:10.3390/ijerph17145009)
Supplement: Supplementary file 1 [file ijerph-17-05009-s001.zip › Table S3-Interview Guide.pdf]

Table S3: Interview Guide

In-depth interview guideline

Date of interview:	
Interview start time:	
Participant’s code:	
Interviewer:	

When dual-method use was/has been practiced.

Topic Focus	Core questions	Additional questions or prompts
**Reasons and motivations for dual-method use**	Why and how do you practice dual-method use with your spouse or live-in partner?	Why do you use condoms with your spouse or live-in sexual partner even when you are using other forms of family planning methods?<br><br>What are barriers/problems to using condoms with other family planning methods (dual-method use) with your spouse or live-in sexual partner?<br><br>What kinds problems/challenges did/do you have?<br><br>- In terms of access to family planning and condoms<br>- In terms of communication with your partner<br>- In terms of social stigma about condom use<br>- In terms of side effects etc.<br><br>Have these challenges been overcome? If yes, how did you overcome these challenges?
**Negotiation and communication for condom use**	How do you communicate/negotiate with your partner about condom use?	Who decided to use family planning and why?<br><br>When did you or/and your partner deicide to use family planning?<br><br>Have you ever discussed family planning with your partner, other family members, health workers etc.? If yes, how often do you discuss with them?<br><br>Who normally decide to use condoms and why?<br><br>Have any risks been considered or discussed with your partner? What risks?<br><br>Do you find it easy to discuss the issue of protection with your partner? Who led the discussion?

**Perceptions of condom use and contraception**	What do you think about using condoms with your partner?	What has your use of condoms with other family planning methods been like with your partner?<br><br>After you started using family planning methods, have you seen any changes in your perception about condom use?<br><br>After you started using family planning methods, have you seen any changes in your partner's perception about condom use?<br><br>Whose responsibility is protection about HIV/STIs? Why?<br><br>Whose responsibility is protection about unintended pregnancies? Why?<br><br>How do you feel about not using protection for HIV/STIs? How does your partner feel?<br><br>Have any actions been taken when protection wasn't used (if there was such a case)?
**Risk perceptions for unintended pregnancy and HIV/STIs**	To what extent do you think about any risks involved with sex?<br><br>What action do you take in relation to these risks?	Do you consider yourself to be at risk of pregnancy? Why? Why not?<br><br>- Have you ever been pregnant? What happened? What did you do? How did you feel? How did other people react? Has it changed your behavior?<br><br>- How would you feel if you found out you were pregnant? What would you do? Why?<br><br>Do you consider yourself to be at risk of HIV/STIs? Why? Why not?<br><br>- Have you had a STI or symptom? Have your partner had a STI or symptom? What happened? What did you do? How did you feel? Has it changed your behavior?<br><br>- Have you ever had an HIV test? Have any of your partners had an HIV test? Why? Why not?<br><br>- Have you ever asked a partner to have a test? Why? Why not?<br><br>- Have you ever had multiple sex partners? Have you ever been paid for sex? Do you use protection during these encounters? Why? Why not?

	Which forms of protection against pregnancy and STIs have you used?<br><br>What, why, when and how was its use or non-use decided?	Which family planning method have you used/do you use? Since when? Why? Where were/are the methods obtained from?<br><br>Have you used condoms throughout the years? Why? Why not?<br><br>How do you decide when to use them?<br><br>Under what circumstances do you/ would you not use them (if there was such a case)? Why? Reasons for engaging in unprotected sex.
**Knowledge of dual-method use**	To what extent do you know about dual-method use?	Do you know family planning methods are not 100% protection against pregnancy?<br><br>Have you ever considered using condoms to prevent pregnancy?<br><br>Have you attended family planning counseling?<br><br>Have you ever learnt how to use condoms?<br><br>Have you ever heard of dual-method use?

When dual-method use was/has NOT been practiced.

Topic Focus	Core questions	Additional questions or prompts
**Reasons and motivations for dual-method use**	Why do not you practice dual-method use with your spouse or live-in partner?	Why do not you use condoms with your spouse or live-in sexual partner even though you are using other forms of family planning methods?<br><br>What are barriers/problems to using condoms with other family planning methods (dual-method use) with your spouse or live-in sexual partner?<br><br>What kinds problems/challenges did/do you have?<br><br>- In terms of access to family planning and condoms<br>- In terms of communication with your partner<br>- In terms of social stigma about condom use<br>- In terms of side effects etc.<br><br>Have these challenges been overcome? If yes, how did you overcome these challenges?

**Negotiation and communication for condom use**	How do you communicate/negotiate with your partner about condom use?	Who decided to use family planning and why?<br><br>When did you or/and your partner deicide to use family planning?<br><br>Have you ever discussed family planning with your partner, other family members, health workers etc.? If yes, how often do you discuss with them?<br><br>Who normally decide to use condoms and why?<br><br>Do you or your partner influence when they are/are not used?<br><br>Have any risks been considered or discussed with your partner? What risks? How were they discussed?<br><br>Do you find it easy to discuss the issue of protection with your partner? Who led the discussion?
**Perceptions of condom use and contraception**	What do you think about using condoms with your partner?	Have you ever used condoms with other family planning methods been like with your partner? When and why? What do you think about it?<br><br>After you started using family planning methods, have you seen any changes in your perception about condom use?<br><br>After you started using family planning methods, have you seen any changes in your partner's perception about condom use?<br><br>Whose responsibility was/is protection about HIV/STIs? Why?<br><br>Whose responsibility was/is protection about unintended pregnancies? Why?<br><br>How do you feel about not using protection for HIV/STIs? How does your partner feel?<br><br>Have any actions (been) taken when protection was not used?
**Risk perceptions for unintended pregnancy and HIV/STIs**	To what extent do you think about any risks involved with sex?<br><br>What action do you take in relation to these risks?	Do you consider yourself to be at risk of pregnancy? Why? Why not?<br><br>- Have you ever been pregnant? What happened? What did you do? How did you feel? How did other people react? Has it changed your behavior?<br><br>- How would you feel if you found out you were pregnant? What would you do? Why?<br><br>Do you consider yourself to be at risk of HIV/STIs? Why? Why not?

		- Have you had a STI or symptom? Have your partner had a STI or symptom? What happened? What did you do? How did you feel? Has it changed your behavior?<br><br>- Have you ever had an HIV test? Have any of your partners had an HIV test? Why? Why not?<br><br>- Have you ever asked a partner to have a test? Why? Why not?<br><br>- Have you ever had multiple sex partners? Have you ever been paid for sex? Do you use protection during these encounters? Why? Why not?
	Which forms of protection against pregnancy and STIs have you used?	Which family planning method have you used/do you use? Since when? Why? Where were/are the methods obtained from?<br><br>Have you used condoms throughout the years? Why? Why not?
	What, why, when and how was its use or non-use decided?	How do you decide when to use them?<br><br>Under what circumstances do you/ would you use them (if there was such a case)? Why? Reasons for engaging in protected sex?
**Knowledge of dual-method use**	To what extent do you know about dual-method use?	Who decided to use family planning and why?<br><br>When did you or/and your partner deicide to use family planning?<br><br>Have you ever discussed family planning with your partner, other family members, health workers etc.? If yes, how often do you discuss with them?<br><br>Who normally decide to use condoms and why?<br><br>Have any risks been considered or discussed with your partner? What risks?<br><br>Do you find it easy to discuss the issue of protection with your partner? Who led the discussion?

Interview end time:	
