# Supplementary material for: Positive Deviance for Dual-Method Promotion among Women in Uganda: A Qualitative Study
Source: Int J Environ Res Public Health. 2020 Jul 12;17(14):5009. doi: 10.3390/ijerph17145009 (PMC7400262; doi:10.3390/ijerph17145009)
Supplement: Supplementary file 1 [file ijerph-17-05009-s001.zip › Table S4-Consolidated criteria for reporting qualitative studies (COREQ) 32-item checklist.pdf]

Table S4- Consolidated criteria for reporting qualitative studies (COREQ): 32-item checklist

Title: Positive deviance for dual-method promotion among women in Uganda: A qualitative study

Authors: Hodaka Kosugi, Akira Shibanuma, Junko Kiriya, Ken Ing Cherng Ong, Stephen Mucunguzi, Conrad Muzoora and Masamine Jimba

(Developed from:Tong A, Sainsbury P, Craig J. Consolidated criteria for reporting qualitative research (COREQ): a 32-item checklist for interviews and focus groups. International Journal for Quality in Health Care. 2007. Volume 19, Number 6: pp. 349 – 357)

No. Item	Guide questions/description	Response/Reported on Page #
**Domain 1: Research team and reflexivity**		
Personal Characteristics		
1. Interviewer/facilitator	Which author/s conducted the interview or focus group?	N/A
2. Credentials	What were the researcher's credentials? E.g. PhD, MD	HK: MA<br>AS: MA, PhD, Assistant Professor<br>JK: MA, PhD, Assistant Professor<br>KICO: MA, PhD, Assistant Professor<br>SM: MD, MPH<br>CM:MD, Sen. Lecture<br>MJ: MD, MPH, PhD, Professor, and Chair
3. Occupation	What was their occupation at the time of the study?	P.1
4. Gender	Was the researcher male or female?	Six males and one female
5. Experience and training	What experience or training did the researcher have?	All the researchers have done previous several qualitative research projects.
Relationship with participants		
6. Relationship established	Was a relationship established prior to study commencement?	See the manuscript in the method section-participant enrolment (P.3-4)
7. Participant knowledge of the interviewer	What did the participants know about the researcher? e.g. personal goals, reasons for doing the research	See the manuscript in the method section-participant enrolment (P.3-4)
8. Interviewer characteristics	What characteristics were reported about the inter viewer/facilitator? e.g. Bias, assumptions, reasons and interests in the research topic	See the manuscript in the method section-participant enrolment (P.3-4)
**Domain 2: study design**		
Theoretical framework		
9. Methodological orientation and Theory	What methodological orientation was stated to underpin the study? e.g. grounded theory, discourse analysis, ethnography,	See the manuscript in the method section/ project

	phenomenology, content analysis	overview and study setting (P.3)
Participant selection		
10. Sampling	How were participants selected? e.g. purposive, convenience, consecutive, snowball	See the manuscript in the method section-participant enrolment (P.3-4)
11. Method of approach	How were participants approached? e.g. face-to-face, telephone, mail, email	See the manuscript in the method section-participant enrolment (P.3-4)
12. Sample size	How many participants were in the study?	See the manuscript in the method section/ study participants (P.3)
13. Non-participation	How many people refused to participate or dropped out? Reasons?	No one has dropped out
Setting		
14. Setting of data collection	Where was the data collected? e.g. home, clinic, workplace	See the manuscript in the method section-data collection and ethics (P.4)
15. Presence of non-participants	Was anyone else present besides the participants and researchers?	See the manuscript in the method section-data collection and ethics (P.4)
16. Description of sample	What are the important characteristics of the sample? e.g. demographic data, date	See the manuscript in the result section/participant characteristics (P.5)
Data collection		
17. Interview guide	Were questions, prompts, guides provided by the authors? Was it pilot tested?	See the manuscript in the method section/ data collection (P.4) and supplementary file 1
18. Repeat interviews	Were repeat inter views carried out? If yes, how many?	No
19. Audio/visual recording	Did the research use audio or visual recording to collect the data?	See the manuscript in the method section/ data collection (P.4)
20. Field notes	Were field notes made during and/or after the interview or focus group?	No
21. Duration	What was the duration of the interviews or focus group?	See the manuscript in the method section/ data collection (P.4)
22. Data saturation	Was data saturation discussed?	No
23. Transcripts returned	Were transcripts returned to participants for comment and/or correction?	No
**Domain 3: analysis and findings**		
Data analysis		
24. Number of data coders	How many data coders coded the data?	Two. See the manuscript in the method section/ data analysis (P.4)
25. Description of the coding tree	Did authors provide a description of the coding tree?	No
26. Derivation of themes	Were themes identified in advance or derived from the data?	See the manuscript in the method section/ data collection

		(P.4)
27. Software	What software, if applicable, was used to manage the data?	See the manuscript in the method section/ data analysis (P.4)
28. Participant checking	Did participants provide feedback on the findings?	No
Reporting		
29. Quotations presented	Were participant quotations presented to illustrate the themes/findings? Was each quotation identified? e.g. participant number	Yes, the interview guide was pretested with five women purposively selected.
30. Data and findings consistent	Was there consistency between the data presented and the findings?	Yes. See the manuscript in the results (P.5-11)
31. Clarity of major themes	Were major themes clearly presented in the findings?	Yes. See the manuscript in the results (P.5-11)
32. Clarity of minor themes	Is there a description of diverse cases or discussion of minor themes?	Yes. See the manuscript in the results (P.5-11)
