# Supplementary material for: Positive Deviance for Dual-Method Promotion among Women in Uganda: A Qualitative Study
Source: Int J Environ Res Public Health. 2020 Jul 12;17(14):5009. doi: 10.3390/ijerph17145009 (PMC7400262; doi:10.3390/ijerph17145009)
Supplement: Supplementary file 1 [file ijerph-17-05009-s001.zip › Table S5-The detailed characteristics of participants included in in-depth interviews.pdf]

Table S5- The detailed characteristics of participants included in in-depth interviews

	Facility	ID	Age	Education	Religion	Occupation	Wealth index	Polygamy	Unintended pregnancy	# of children	Pregnancy intention	Partner's Pregnancy intention	HEC type	Frequency of condom use	History of HIV/STIs
PD	1	22	40	Never	Muslim	Agriculture	Poorer	No	Yes	8	No	Don't know	Injectables	Every time	No
	2	2	18	Never	Roman catholic	Unemployed	Poorer	No	No	1	Yes	Yes	Injectables	Every time	No
	2	4	27	Never	Protestant/other Christian	Own business	Poorer	No	No	2	Yes	No	Injectables	Every time	No
	2	14	25	Secondary or more	Protestant/other Christian	Own business	Richest	No	Yes	1	Yes	Yes	Injectables	Every time	No
	3	24	31	Primary	Protestant/other Christian	Own business	Richer	Yes	Yes	3	Yes	Yes	Injectables	Every time	No
	4	10	27	Primary	Roman catholic	Own business	Richest	No	No	3	Yes	Yes	OCPs	Every time	Yes
	5	4	22	Primary	Roman catholic	Private sector	Poorest	No	No	1	Yes	Yes	Implants	Every time	No
	5	21	21	Primary	Muslim	Unemployed	Poorest	No	No	1	Yes	Yes	Injectables	Every time	No
Non-PD	1	7	28	Primary	Roman catholic	Agriculture	Middle	No	Yes	3	Yes	Yes	Injectables	Never	No
	1	25	36	Primary	Protestant/other Christian	Own business	Poorest	No	Yes	5	Undecided	Don't know	Injectables	Never	No
	2	21	23	Never	Muslim	Unemployed	Poorest	No	No	2	Undecided	Don't know	Implants	Never	No
	2	29	27	Primary	Protestant/other Christian	Agriculture	Richer	No	No	2	Yes	Don't know	Injectables	Never	Yes
	3	19	38	Primary	Roman catholic	Own business	Richer	No	Yes	5	No	No	Injectables	Never	Yes
	3	28	27	Primary	Protestant/other Christian	Own business	Richer	Yes	Yes	2	No	Don't know	Implants	Never	Yes
	4	6	34	Primary	Protestant/other Christian	Own business	Middle	No	No	2	No	No	Injectables	Never	No
	4	30	27	Primary	Protestant/other Christian	Own business	Richest	No	No	1	Yes	Yes	Implants	Never	Yes
	5	8	29	Primary	Protestant/other Christian	Own business	Poorest	No	No	3	Yes	Yes	Injectables	Never	Yes
	5	17	24	Primary	Protestant/other Christian	Private sector	Poorest	Yes	No	1	Yes	Yes	Injectables	Never	No

PDs: Positive Deviants; HIV: Human Immunodeficiency Virus; STIs; sexually transmitted infections; HECs: Highly Effective Contraceptives; IUD: Intrauterine Device; OCPs: oral contraceptive pills.
