# Supplementary material for: Positive Deviance for Dual-Method Promotion among Women in Uganda: A Qualitative Study
Source: Int J Environ Res Public Health. 2020 Jul 12;17(14):5009. doi: 10.3390/ijerph17145009 (PMC7400262; doi:10.3390/ijerph17145009)
Supplement: Supplementary file 1 [file ijerph-17-05009-s001.zip › Text S6-Barriers to dual-method use which were not directly related to HEC use for women in stable relationships.pdf]

Supplementary File 6：Barriers to dual-method use which were not directly related to HEC use for women in stable relationships

We present barriers which were not directly related to HEC use as supplementary information of the results reported in the main text.

1. Low perceived need for dual-method use due to HIV-negative status

Four non-PDs mentioned that they had tested negative for HIV as a reason for not using condoms. The majority of both PDs and non-PDs knew their partner's HIV status and had undergone testing either with their partners or separately in the past.

"We went through HIV-testing before when I delivered our last born, and we know that we are both negative. I understand that condoms are necessary to prevent HIV infection. But I do not think married couples like us need it."

(Non-PD, 27 years old, 4-030)

Another non-PD mentioned:

"I can enjoy sex without using condoms and still prevent unplanned pregnancies thanks to implants. I obtain more satisfaction when I don't use them. Condoms are even costly for nothing, yet family planning is provided for free."

(Non-PD, 27 years old, 3-028)

2. Difficulty in suggesting and persuading partners to use condoms in marital relationships

Some non-PDs had never discussed condom use with their partners, although they felt that they were at risk of STIs and had used condoms before. Their silence on the subject was due to their fear of rejection and their unwillingness to raise distrust and a sense of awkwardness by introducing the topic of condoms in their marriage.

"Many men are cheating on their wives, and it is normal because we are all human. Some men, including my husband, did not agree to go to HIV testing with their wives. Condoms are important, but I have never discussed it with my husband as we are married."

(Non-PD, 24 years old, 1-007)

Another non-PD mentioned:

"I fear suggesting condoms because I do not know how my partner will react. He may think I am suspecting that he is doing something bad with other girls and get mad at me. He may also suspect I have a disease. I do not want such troubles."

(Non-PD, 27 years old, 2-029)

Several PDs and non-PDs had experienced objection from their partners. Non-PDs had been discouraged from suggesting condom use again after meeting with opposition from their partners. One non-PD talked about her struggle to persuade her partner which had ended when her partner became violent over the matter. She tried to introduce condom use with her partner after developing what she suspected was an STI.

"I got candida before, and I am sure it came from my partner. After I went to the clinic, I suggested that he use a condom. But he did not admit that he had infected me and refused to use condoms. I could not talk about it as he seemed really annoyed."

(Non-PD, 38 years old, 3-019)

3. Access to condoms

Although condoms were provided free of charge at health centers, they were not always available for the women in this study due to financial and psychological barriers and insufficient supply. In total, five PDs and four non-PDs had experienced that they were unable to obtain condoms when they needed them. One non-PD mentioned that her low household income was a barrier to buying condoms. She stated that she had to prioritize other household expenditures over condoms.

"I do not have regular work that is why I am not able to buy condoms. No one will buy condoms over food when they have nothing to feed themselves and their children."

(Non-PD, 23 years old, 2-021)

Another non-PD mentioned that her partner normally obtained the condoms she had used in the past. When women relied on their male partners to provide condoms, they might have difficulty in obtaining them. The non-PD described this situation as follows:

"My husband had brought condoms from somewhere, and we have used them several times. But he did not have one when I suggested condom use. I did not know if he was just telling me a lie or did not really have any."

(Non-PD, 34 years old, 4-006)

Three PDs and two non-PDs expressed their concerns about obtaining condoms from dispensers in public health facilities because of their shyness and the large number of patients who may see them receiving condoms. A non-PD mentioned this:

"There are always many patients in the health center. If they see me receiving condoms, they will suspect that I have HIV or that I am cheating on my husband. Many people know I am already married and have three children. I do not want to feel ashamed because of that."

(Non-PD, 29 years old, 5-008)

Three PDs and one non-PD also said that the health centers normally provided condoms free of charge but sometimes ran out of condoms.

[Checked up to here last time]

4. Concerns about condom use

Another common issue was the poor quality of condoms. Three PDs and two non-PDs reported that they disliked condoms because of their unpleasant rubber-and-lubricant smell and because of the itchiness and rashes that they can cause both for themselves and for their partners. One PD and two non-PDs expressed their concerns about the breakage of condoms. One non-PD mentioned that she did not use a condom because of her fear based on the myth that condom use can cause cancers.

"My partner did not agree to use condoms as they had caused itchiness and rashes before. He dislikes it. I also heard that condoms could cause cancer in women, so I prefer to rely on injections."

(Non-PD, 24 years old, 5-017)

Some PDs and non-PDs also reported a reduction in their sexual urge and satisfaction as a problem caused by using a condom.
